# Plated Cambrian Bilaterians Reveal the Earliest Stages of Echinoderm Evolution

**DOI:** 10.1371/journal.pone.0038296

**Published:** 2012-06-06

**Authors:** Samuel Zamora, Imran A. Rahman, Andrew B. Smith

**Affiliations:** 1 Department of Palaeontology, The Natural History Museum, London, United Kingdom; 2 School of Geography, Earth & Environmental Sciences, University of Birmingham, Edgbaston, Birmingham, United Kingdom; Brigham Young University, United States of America

## Abstract

Echinoderms are unique in being pentaradiate, having diverged from the ancestral bilaterian body plan more radically than any other animal phylum. This transformation arises during ontogeny, as echinoderm larvae are initially bilateral, then pass through an asymmetric phase, before giving rise to the pentaradiate adult. Many fossil echinoderms are radial and a few are asymmetric, but until now none have been described that show the original bilaterian stage in echinoderm evolution. Here we report new fossils from the early middle Cambrian of southern Europe that are the first echinoderms with a fully bilaterian body plan as adults. Morphologically they are intermediate between two of the most basal classes, the Ctenocystoidea and Cincta. This provides a root for all echinoderms and confirms that the earliest members were deposit feeders not suspension feeders.

## Introduction

Echinoderms are the animal phylum that has departed most radically from the ancestral bilaterian body plan [1], [2]. Whereas other higher metazoans all share a body plan that is basically bilaterally symmetrical, echinoderms are constructed with a pentaradial arrangement of appendages that makes them instantly recognizable but raises major problems for homologizing their body axes with those in other phyla [3]. Molecular data show that the sister group to echinoderms are the hemichordates [4]–[7], a clade that includes both the deposit feeding enteropneusts and suspension feeding pterobranchs. However, this helps little in our understanding of the origin of the echinoderm body plan or the mode of life of the earliest echinoderms. Hemichordates are bilaterally symmetric, but so different morphologically from echinoderms that few characters other than those shared by all deuterostomes can be homologized, making them a poor outgroup for rooting the echinoderm tree [7]. Furthermore, there is uncertainty as to whether pterobranchs are sister group to enteropneusts or a derived clade nested within enteropneusts [2], [8], [9], making the ancestral body plan of hemichordates ambiguous. However, clues to the origins of the echinoderm body plan organization can be gained from the ontogeny of extant taxa: an initially bilaterally symmetrical larva undergoes an asymmetric metamorphosis that involves a complete body-axis shift, eventually giving rise to a pentaradiate adult with five ambulacral areas [10], [11]. In effect, the adult pentaradiality of ambulacral rays is derived ultimately from elaboration of a single larval coelom, originally one of a pair. This implies that echinoderm evolutionary history proceeded first through a bilateral and then an asymmetrical phase before arriving at the ubiquitous pentaradiate morphology shown by all crown group echinoderms.

**Figure 1:**
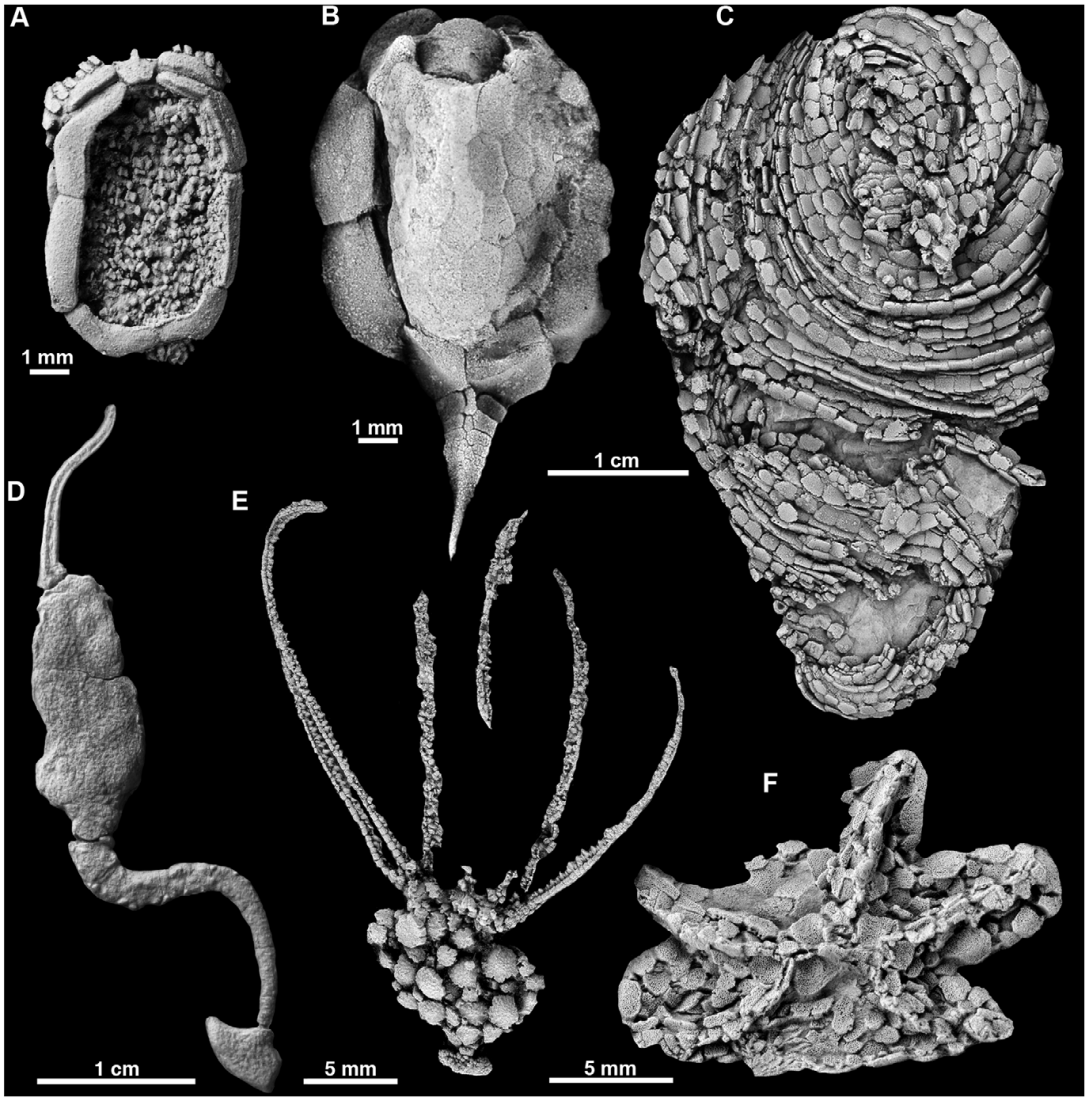
Radiate and asymmetric echinoderms from the Cambrian showing a selection of primitive echinoderm body plans. **A**, the ctenocystoid *Ctenocystis*; **B**, the cinctan *Gyrocystis*; **C**, the helicoplacoid *Helicoplacus*; **D**, the solute *Coleicarpus*; **E**, the eocrinoid *Gogia*; **F**, stromatocystitid edrioasteroid.

Fortunately, echinoderms have left behind an excellent fossil record that illuminates some of the initial steps involved in the assembly of their unique body plan [12]. Fossil echinoderms from the Cambrian include both radiate and asymmetric forms [13], [14] (Figure 1). Their identity as total group echinoderms is in no doubt because all possess a skeleton composed of stereom, an autapomorphy for the clade [15]. The radiate forms (e.g. helicoplacoids, stromatocystitids, gogiids) have between two and five primary ambulacral rays. Because only a single asymmetrically-placed hydropore is ever present in these forms and their ambulacral construction is closely similar, we deduce that their water vascular system must be like that of extant echinoderms and elaborated from a single coelom. Therefore these are derived morphologies that provide little help in understanding the pre-radial history of echinoderms, although they do reveal the great range of subsequent diversification that took place once radiality had been achieved [16]. More interesting are the echinoderms that show no evidence of radiality and that have long been interpreted as more primitive [12], [17]–[19]. These include forms that have a single asymmetrically positioned ambulacral ray and hydropore (solutes and, according to some interpretations, e.g. [20], stylophoroans), and those with asymmetrically paired marginal grooves and an anterolateral mouth (cintans) [21]. Possibly most basal of all are the weakly asymmetric ctenocystoids [22]. These are almost bilateral in their organization but are constructed with differing numbers of marginal plates on left- and right-hand sides of the body, especially evident in the ventral marginal ring, e.g. [23]. Surprisingly, despite both phylogenetic relationships and the larval development of extant echinoderms pointing to a bilateral ancestry for echinoderms, there has, until now, been no convincing fossil record of this evolutionary stage.

Here we report a new taxon, *Ctenoimbricata* gen. nov., from the earliest middle Cambrian (Cambrian Series 3, Stage 5) of the Iberian Chains, NE Spain, which has a multiplated skeleton with a bilaterally symmetrical construction. This we interpret to be the most basal known echinoderm, closest in morphology to the bilaterian latest common ancestor of all asymmetric and radiate forms. Based on new material of the poorly known genus *Courtessolea* from slightly younger rocks of the Montagne Noire, France, we show in addition that basal ctenocystoids are also bilaterally symmetric.

## Materials and Methods

### Geological Setting and Stratigraphy

**Figure 2:**
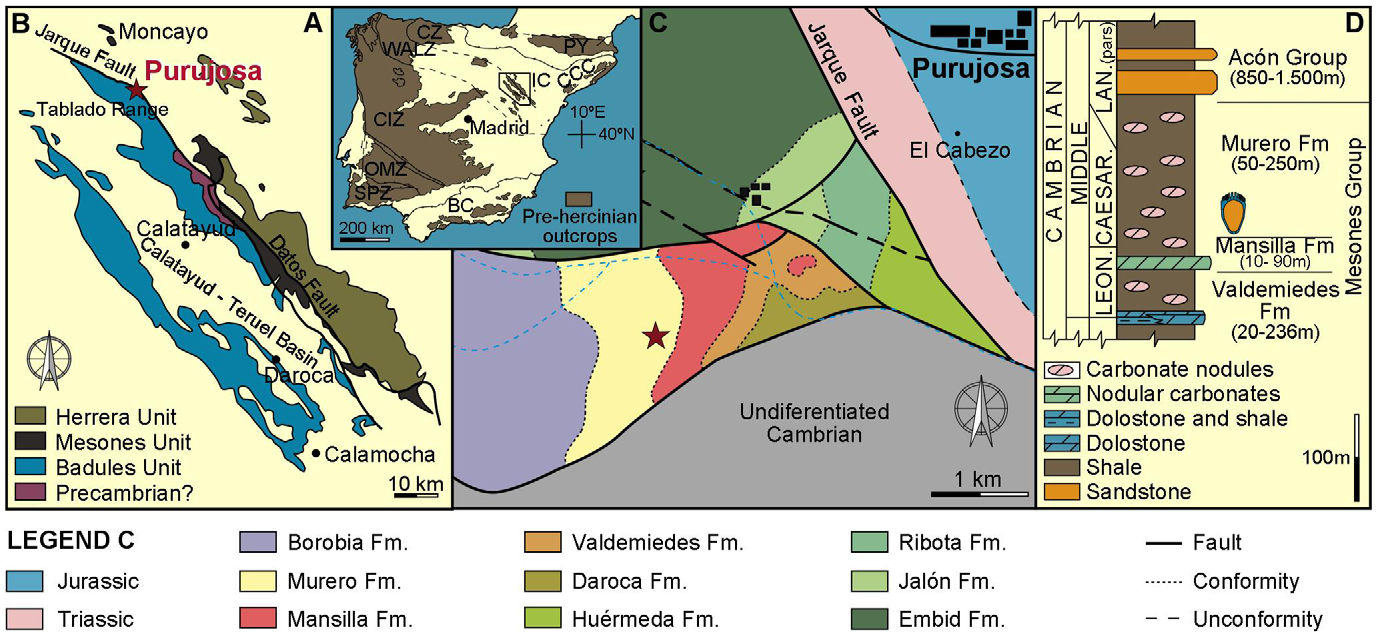
Fossil locality and geological setting. **A**, Map of Spain showing the location of the Iberian Chains (IC). **B**, Map showing the Purujosa locality in the northern part of the Iberian Chains. **C,** Geological map of Purujosa with indication of the studied section (marked with a star). **D**, Composite stratigraphic section indicating the level from where *Ctenoimbricata spinosa* was collected.

*Ctenoimbricata spinosa* gen. et sp. nov. comes from the lowermost part of the Murero Formation at Purujosa, 2 km south of Purujosa village, Moncayo Natural Park, in the northern part of the Iberian Chains, NE Spain (Figure 2). Specimens come from the basal part of section Purujosa 6, which is middle Caesaraugustan in age, and were excavated under permit by the Gobierno de Aragon. The Murero Formation comprises a siliciclastic succession with some interbedded carbonate nodules, and is interpreted as having been deposited during transgressive conditions in an offshore environment. The position of Purujosa in the most distal part of the Iberian Chains favoured the preservation of multiple obrution events in which articulated echinoderms and complete trilobites are common [14], [24]. In addition, a new specimen of the ctenocystoid *Courtessolea moncereti* was collected by Mr. Daniel Vizcaïno from the classic section of Ferrals-les-Montagnes in the Montagne Noire, southern France and comes from the Coulouma Formation, which is Lower Languedocian in age. Both fossil levels fall within Cambrian Series 3, Stage 5 based on chemostratigraphic data from Álvaro *et al*. [25] in the latest Global Stratigraphic System (ca. 510 Ma).

### Imaging Techniques

Two specimens of *Ctenoimbricata spinosa* were imaged using X-ray micro-tomography (µCT). The holotype was scanned on a Metris X-Tek HMX-ST at the Natural History Museum, London; the paratype was scanned on a SkyScan 1172 in the School of Dentistry at the University of Birmingham. The resulting parallel slice images (1200 for the holotype and 819 for the paratype) were independently reconstructed as three-dimensional models using the custom SPIERS software suite [26]. An inverted linear threshold was applied to each dataset in order to create binary images that did not show the matrix surrounding the fossil. Additional objects irrelevant to the fossil were then carefully removed from these thresholded images. Finally, regions-of-interest (i.e. important anatomical characters such as skeletal plates) were manually defined. In most cases, individual plate boundaries could be virtually differentiated based on the presence of sediment infill between plates. However, the ventral integument is obscured by a large crack in both specimens and thus could not be reconstructed in detail. In addition, the dorsal ctenidial plates could not be individually assigned to regions-of-interest because they are very thin and there is no infilling sediment between them to mark boundaries. Multiple isosurfaces representing different regions-of-interest were reconstructed to enable detailed morphological description of the fossils. High-quality ray-traces were produced for these reconstructions using Blender.

Latex casts of the specimens provided additional morphological information. Although not all the important anatomical characters could be studied using this technique, latex casts proved critical for reconstructing the ventral integument. The spinose ctenidial plates forming the distal-most row of the ctenidium were most clearly visible when the holotype was submerged in water.

### Nomenclatural Acts

The electronic version of this document does not represent a published work according to the International Code of Zoological Nomencalture (ICZN), and hence the nomenclatural acts contained in the electronic version are not available under that Code. Therefore, a separate edition of this document was produced by a method that assures numerous identical and durable copies, and those copies were simultaneously obtainable from the publication date noted on the first page of this article for the purpose of providing a public and permanent scientific record, in accordance with Article 8.1 of the Code. The separate print-only edition is available on request from PloS by sending a request to PloS ONE, 1160 Battery Street Suite, San Francisco, CA 94111, USA along with a check for $10 (to cover printing and postage) payable to “Public Library of Science”. Digital archives where the present paper is deposited are PubMedCentral and LOCKSS. In addition, this published work and the nomenclatural acts it contains have been registered in ZooBank, the proposed online registration system for the ICZN. The ZooBank LSIDs (Life Science Identifiers can be resolved and the associated information viewed through any standard web browser by appending the LSID to the prefix http://zoobank.org/. The LSID for this publication is: urn:lsid:zoobank.org:pub:3EB88D62-88FD-40F8-A735-024F8189B21D.

## Results

### Morphology of *Ctenoimbricata*

**Figure 3:**
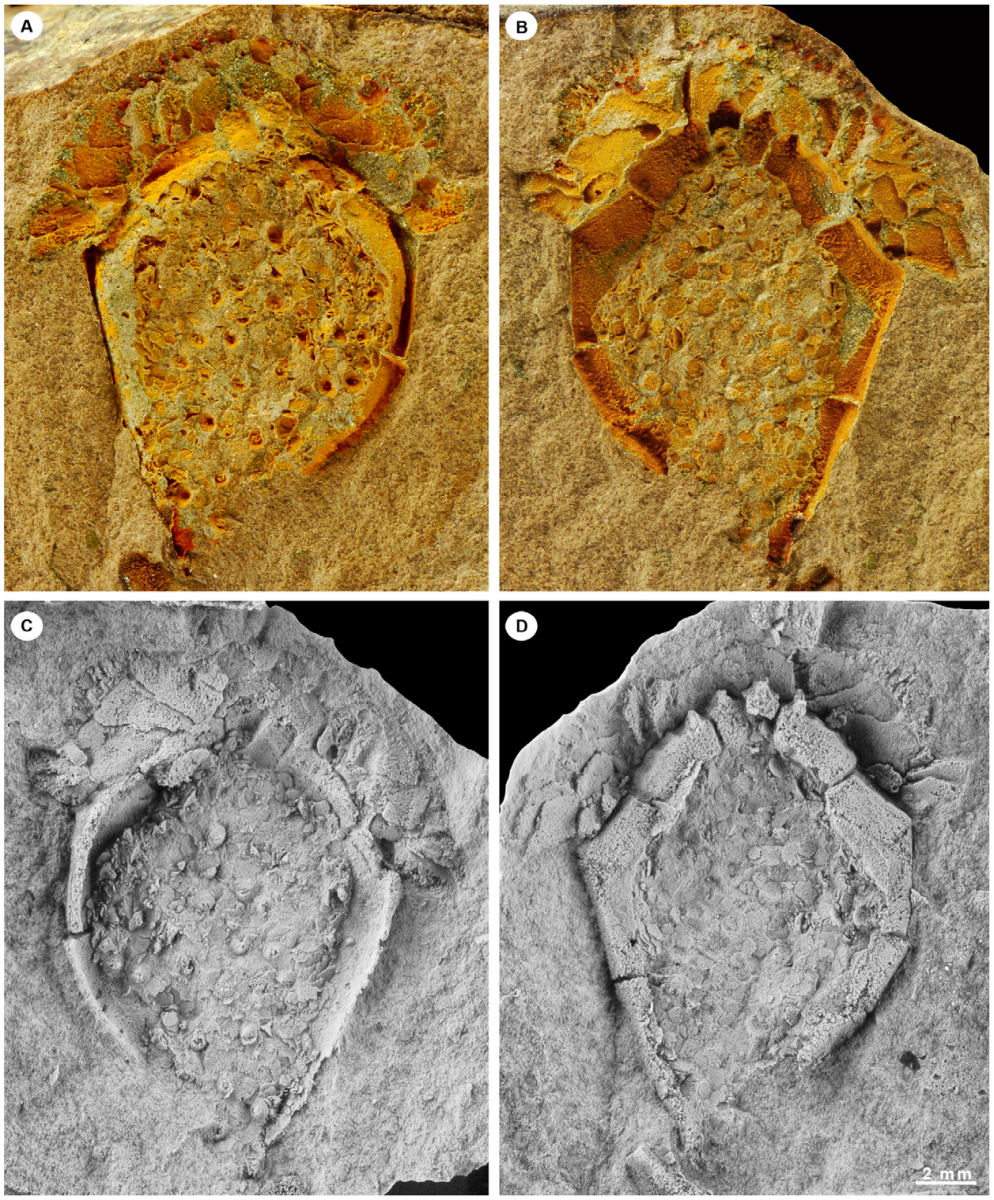
Natural mould and latex casts of the holotype of *Ctenoimbricata spinosa* gen. et sp. nov. (MPZ 2011/93) in dorsal (A, C) and ventral views (B, D). Latex casts were whitened with NH_4_Cl sublimate.

**Figure 4:**
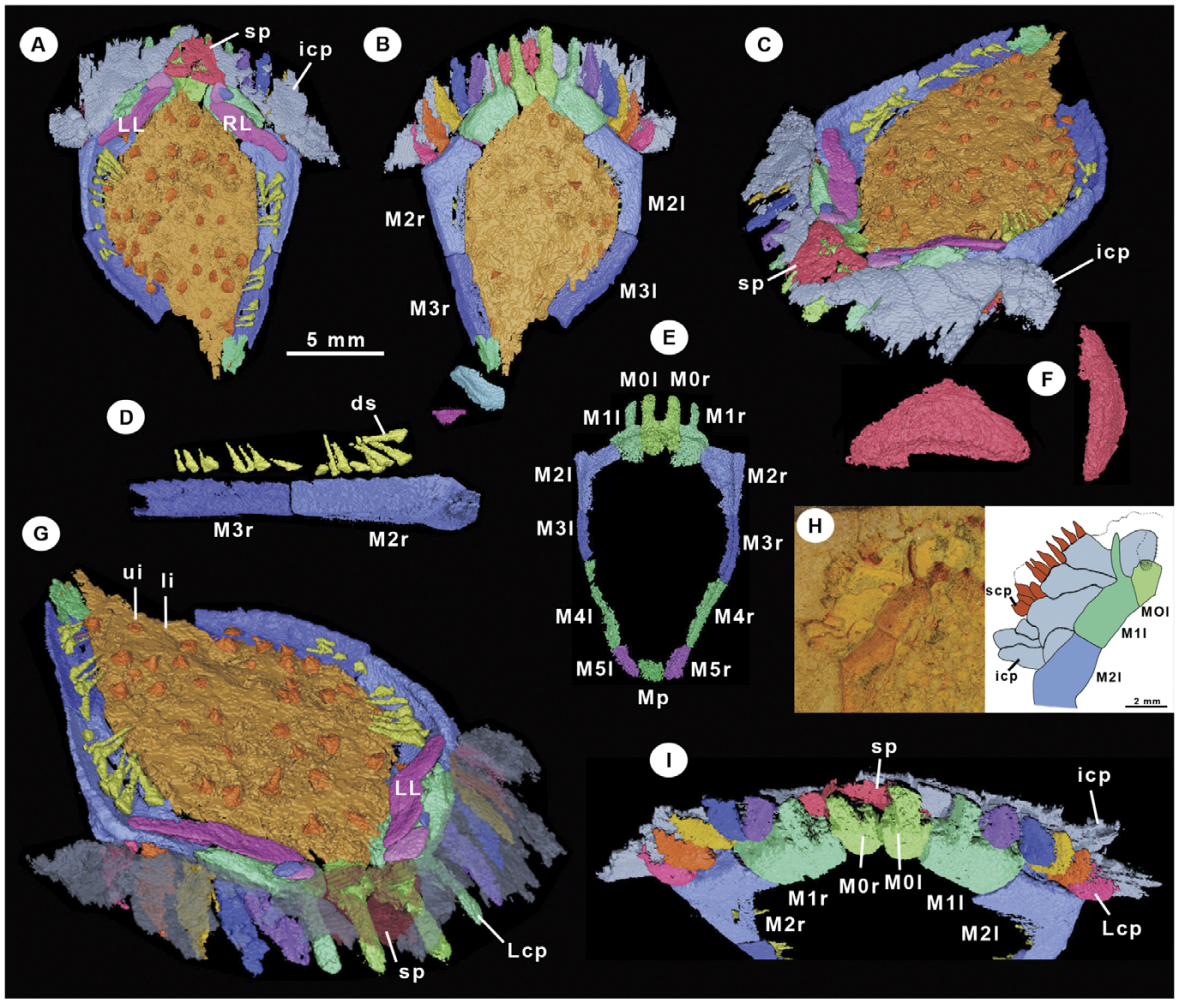
*Ctenoimbricata spinosa* gen. et sp. nov. Cambrian Series 3, Stage 5, Purujosa, Spain. Computer models (**A**–**G**, **I**) and photograph with interpretive camera lucida drawing (**H**). (**A**–**D**, **G**–**I**) Holotype MPZ 2011/93. (**E**, **F**) Paratype MPZ 2011/94. (**A**, **B**) Dorsal and ventral views. (**C**) Oblique left view. (**D**) Lateral view of two marginal plates showing the articulation of the spines. (**E**) Marginal frame plates after correction of plate orientations. (**F**) Suroral plate in dorsal and lateral aspect. (**G**) Oblique right view with the dorsal ctenidium partially transparent to show the ventral ctenidial plates. (**H**) Left anterior part of the theca showing the arrangement of the dorsal ctenidial plates. (**I**) Frontal view. Abbreviations: ds (dorsal spines), icp (imbricate ctenidial plates), Lcp (lower ctenidial plates), LL (adoral left plate), M (marginal plate), RL (adoral right plate), scp (spiny ctenidial plates), sp (suroral plate), ui, li (upper and lower integuments).

**Figure 5:**
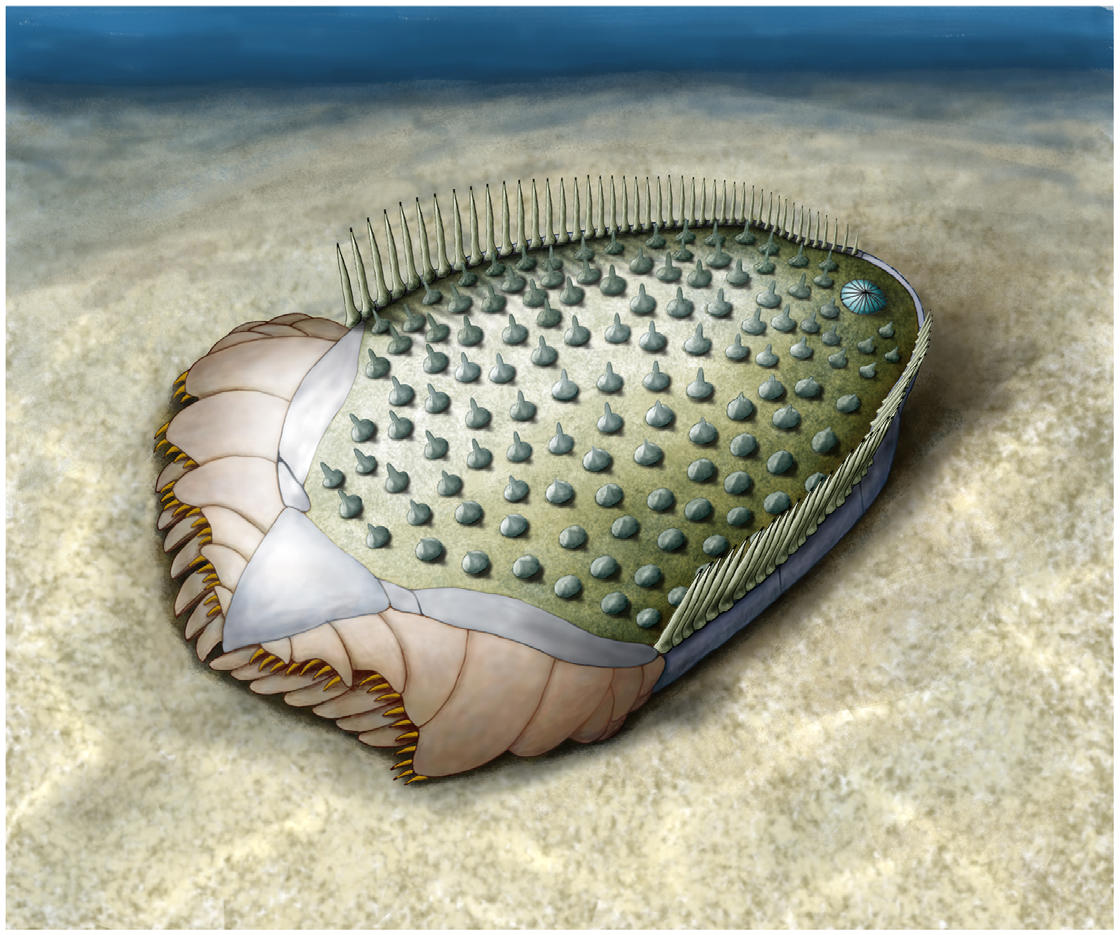
Reconstruction of *Ctenoimbricata spinosa* gen. et sp. nov.

*Ctenoimbricata* is a small (20 mm), disc-like animal with a clearly defined anterior–posterior axis and with skeletal elements arranged bilaterally symmetrically along that axis (Figures 3, 4, 5, Movies S1, S2). A uniserial marginal ring of stout plates frames the body, comprising four elements at the anterior forming part of the ctenidium (Fig. 4E, plates M0, M1), four on either side (plates M2–M5) and a single posterior element (plate Mp). Dorsal and ventral plated membranes cover the centre of the disc. At the anterior, there is a wide opening framed by marginal plates and covered dorsally by a sheet of imbricate plates. This dorsal roof is formed of several superimposed series of thin, flat plates that imbricate to the posterior. A row of very small spinose plates forms the outermost dorsal row. The dorsal ctenidium formed a single unit with limited flexibility. Ventrally, the opening is lined anteriorly by 14 spinose elements. The four median ones are anterior extensions of marginal frame plates M0 and M1. The remaining 10 are free elements that articulate with the outer edge of marginal plates. Distally, these plates taper, becoming knife-like, and they overlap from posterior to anterior. The periproct is not seen but certainly does not pass through the marginal ring, as this is unbroken. It must therefore be situated in the dorsal membrane, and the only part of that structure missing from our specimen is the very posterior. By comparison with *Courtessolea* (see below), therefore, we conclude that the periproct must have opened in the posterior part of the dorsal membrane, close to plate Mp.

### Morphology of *Courtessolea*

**Figure 6:**
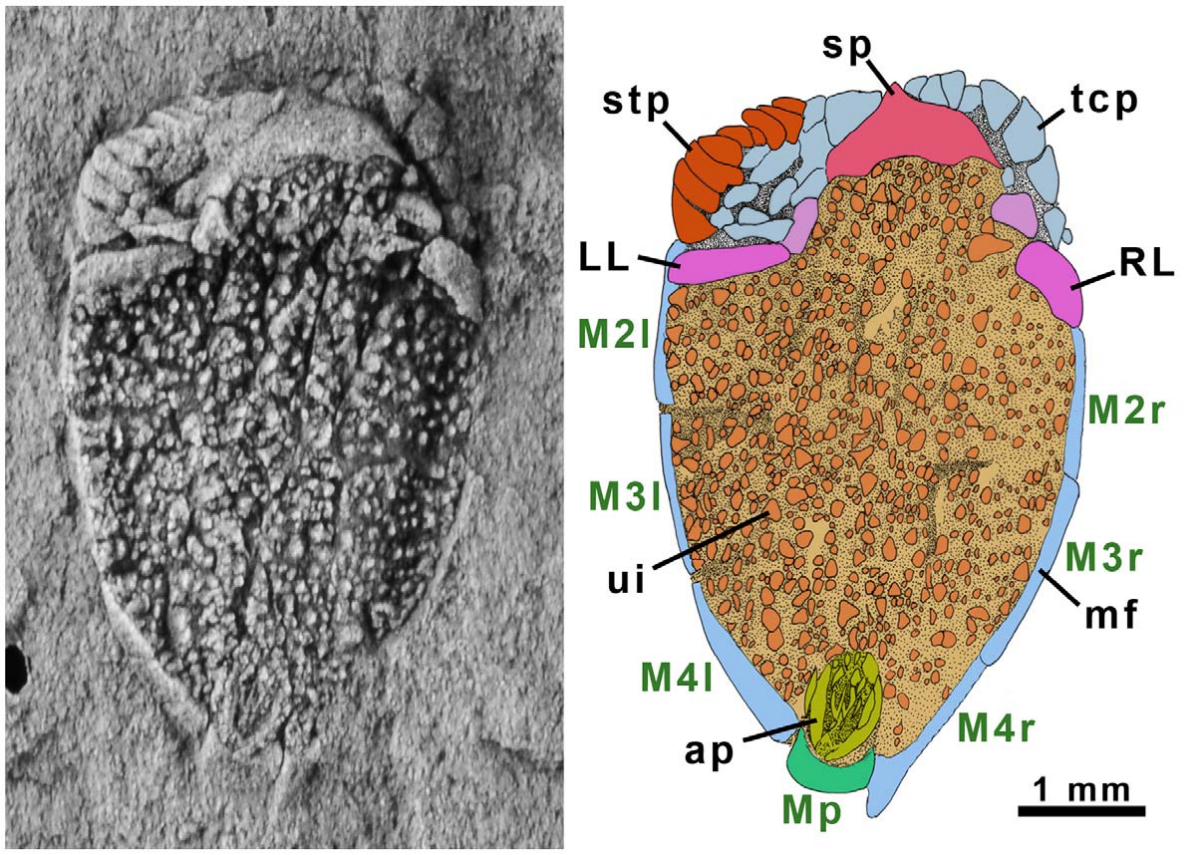
*Courtessolea moncereti* Domínguez-Alonso, 1999 (MNHN F.A45783). Cambrian Series 3, Stage 5, Ferrals-les-Montagnes, France. Dorsal view and interpretive diagram (note that the theca shows a small degree of post-mortem distortion). Abbreviations: ap (anal pyramid), LL (adoral left plate), M (marginal plate), mf (marginal frame), Mp (posterior marginal plate), RL (adoral right plate), stp (spiny ctenidial plates), sp (suroral plate), tcp (tessellate ctenidial plates), ui (upper integument).

*Courtessolea* (Figure 6) is similar to *Ctenoimbricata*, except in having a complete dorsal and ventral ctenidium of large spinose plates and a much-reduced band of tessellate plates dorsally (probably homologous with the imbricate roof of *Ctenoimbricata*). It is bilaterally symmetrical with a single ring of marginal frame plates, and its periproct opens through the dorsal plated membrane immediately in front of the posterior marginal plate and on the anterior-posterior axis. There are four marginal plates at the anterior forming part of the ctenidium, three marginal plates (M2, M3, M4) along each side and a single plate bounding the tapering posterior part (Mp).

### Homologies Shared Amongst Primitive Echinoderms

**Figure 7:**
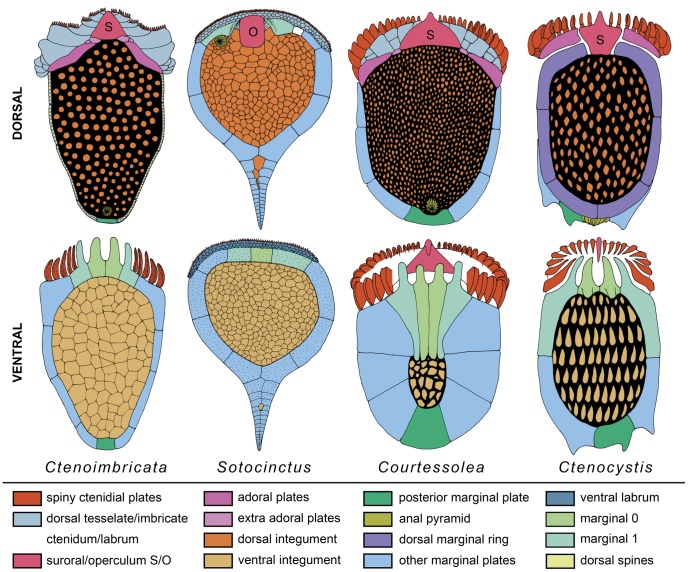
Diagram showing inferred homologies between ctenocystoids (*Ctenocystis* and *Courtessolea*), *Ctenoimbricata* and cinctans (*Sotocinctus*). The upper row illustrates dorsal surfaces, the lower row ventral surfaces; colors indicate plating series that are homologized. Reconstructions of ctenocystoids are modified from [34]. S  =  suroral plate; O  =  operculum.

Figure 7 summarizes the homologies that we draw between cinctans, ctenocystoids (*Ctenocystis*, *Courtessolea*) and *Ctenoimbricata*. Three openings are identified in cinctans [21], but only two in ctenocystoids [23] and *Ctenoimbricata*. Whereas cinctans have two anterior openings close together, identified as exhalant (atrial) and inhalant (mouth) orifices [12], [21], [27], *Ctenoimbricata* and ctenocystoids have, in the same position, only one wide opening. This implies that in ctenocystoids and *Ctenoimbricata* the wide anterior opening accommodates both inhalant and exhalant flows and has the combined function of feeding and expelling water from the interior of the theca. In cinctans this flow has become partitioned and the left-hand inhalant flow channeled to the mouth (on the animal’s right-hand side) via the anterior groove. Ctenocystoids and cinctans are interpreted as pharyngeal basket feeders with internal gill slits, akin to tunicates [10], [23] and the same is likely true for *Ctenoimbricata*.

Homologies of the anterior elements are clearest between ctenocystoids and *Ctenoimbricata.* The ventral anterior surface is constructed in both of four marginal plates (M0 and M1) with spinose elements that contribute to the ctenidium and which are clearly homologous. In *Ctenocystis* plate M1 extends laterally around the cinctus whereas in *Courtessolea* and *Ctenoimbricata* it is plate M2 that forms the anterior border. On the dorsal side all have a large, central, suroral plate that is triangular and thin in *Courtessolea* and *Ctenoimbricata* but more robust in *Ctenocystis*. The dorsal imbricate plate series seen in *Ctenoimbricata* is also present but reduced and tessellate in *Courtessolea* and the distal spinose elements enlarged and probably homologous to the much larger ctenoid plates in *Ctenocystis*. Dorsal imbricate plates are lacking in *Ctenocystis* and the ctenoid elements rest directly on marginal frame plates or on the adorals. Finally, in *Ctenoimbricata* and *Courtessolea* marginal frame elements are bilaterally arranged with a median posterior element whereas they are asymmetrical in *Ctenocystis*.

Homologies of the anterior plating between *Ctenoimbricata* and cinctans are less apparent but can still be traced. Most obvious is the similarity between the operculum of cinctans and the large suroral plate of *Ctenoimbricata*, which in both cases covers the central part of the anterior opening and is presumably associated with controlling currents into or out of the theca. At least part of the ctenidium is homologous with the labrum in cinctans. In the most primitive cinctans the labrum is composed of several rows of tessellate plates with the most distal row being spinose [27]. The same arrangement is present dorsally in *Ctenoimbricata* although the plates there are much larger. Ventral ctenidial plating in *Ctenoimbricata* is more difficult to homologize. The two most anterior plates in the marginal frame of *Ctenoimbricata* are most likely homologous with the single M0 of cinctans because in both cases they floor the oral plate/operculum and create a large embayment to the anterior opening. The shift from two plates in ctenocystoids to a single plate in cinctans involved either the fusion of these plates or the loss of one.

### Systematic Palaeontology

#### Nomenclature

Here we follow the orientation proposed by Robison and Sprinkle [22] and Sprinkle and Robison [28] for ctenocystoids, with upper/superior equivalent to dorsal. The system for naming plates, however, has been modified in light of the established plating homologies with cinctans (Fig. 7). Rahman and Clausen [23] used the terms RSb and LSb to refer to the two most anterior ventral plates that contribute to the ctenidium in *Ctenocystis*. These plates are part of the marginal frame in *Courtessolea* and probably homologous with plate M0 of cinctans (Fig. 7). These two plates in ctenocystoids are therefore referred to as M0l and M0r, depending on whether they are positioned to the right (r) or left (l) of the midline. The remaining plates of the marginal frame are then numbered sequentially as M1l to Mnl and M1r to Mnr. While anterior parts should be homologous among ctenocystoid species because all bear the ventral spinose projections from the ventral ctenidium, there are no obvious homologies in the medial part of the ring. The posterior plate of the marginal ring in ctenocystoids does not have a clear equivalent in cinctans and is here referred to as Mp. It could be homologous to any of the most proximal mesosphenoid plates of the cinctan stele (see [21]). While there are obvious homologies in the anterior part of the marginal ring amongst different species, we are not able to recognize homologies in the medial part. The plate Mp is treated as homologous in all ctenocystoids.

Within the ctenoid apparatus, we recognize the suroral plate (dorsal), the left and right adoral plates (dorsal) and several additional adoral plates (dp). The ventral plates of the ctenoid apparatus (the ctenoid plates) are numbered successively along the apparatus from the most adoral to the most aboral as Ct1L, Ct1R, Ct2L, Ct2R, etc., with L and R indicating their position to the left or right of the midline.

Stem group Echinodermata.

*Ctenoimbricata* gen. nov.

urn:lsid:zoobank.org:act:6DD6C927-AE18-4378-A4A1-B24ADAABAD68.

**Type species:** *Ctenoimbricata spinosa* sp. nov.

**Locality and horizon:** As for type species.

**Diagnosis:** As for type species.

*Ctenoimbricata spinosa* sp. nov.

urn:lsid:zoobank.org:act:B1049C1E-4186-4951-8FDF-BC9080B47E0D.

**Types:** Holotype, Zaragoza University Museum MPZ 2011/93; paratype, MPZ 2011/94.

#### Locality and horizon

Purujosa, section 6, Moncayo Natural Park, northernmost Iberian Chains, North Spain. *Pardailhania hispida* Zone, middle Caesaraugustan, Cambrian Series 3, Stage 5.

#### Diagnosis

Theca oval to rhomboid in shape with a single marginal ring of plates bearing spines. Ctenidium composed of a dorsal roof built with a proximal row of imbricate plates and tiny spinose distal plates, and a ventral part composed of 14 spinose elements, of which the four central-most represent projections of marginal plates M0 and M1. Dorsal surface weakly calcified with scattered stud-like ossicles.

### Morphological Description

#### Aperture

A single, wide aperture is located in the anterior part of the body where plates M0 and M1 are embayed. Dorsally, the aperture is covered by the suroral plate and the dorsal ctenidium. The width of the aperture is ca. 4 mm, but its height varies depending on the position of the suroral plate and dorsal ctenidium.

#### General shape

The theca is flattened in profile and bilaterally symmetrical, and is ovoid to sub-rhomboidal in outline. A marginal ring of plates frames the body cavity, which is covered ventrally by a continuous plated ventral membrane and dorsally by a largely uncalcified membrane with some scattered spine-like plates. The ctenidium forms almost the entire anterior border of the theca.

#### Marginal ring

The marginal ring is uniserial and composed of 13 plates (M0r–M5r, M0l–M5l, Mp). M0l and M0r are rectangular, longer than wide, with a large, spinose anterior projection. They abut laterally with plates M1l and M1r respectively, and posteriorly with the ventral integument. They are flat externally, but their internal surface is slightly concave and frames the large anterior opening.

M1l and M1r are rectangular, wider than long, and like M0l and M0r have spinose anterior projections. They have a flat external surface, but have an internal embayment that forms part of the large anterior opening. In cross-section, they have a thick anterior part and a very thin posterior. Along their anterior part are large pits (ligamentary pits) for the articulation of ventral ctenidial plates. Their posterior ventral margin articulates with plates of the ventral integument. The oral opening extends beneath these plates for at least 75% of their length. The posterior dorsal parts articulate with the adoral plates (LL and RL).

M2l and M2r are angled, with the anterior part supporting and contributing to the ctenidium and the posterior part continuous with the marginal ring. Their ventral surface is flat, as is the case for all marginal plates, but their dorsal surface changes: around the anterior it articulates with the adoral ctenidial plates, whereas the posterior part has articulation pits for dorsal spines. In cross-section these plates are triangular with a well-developed internal and ventral flange. At least nine dorsal articulation points are present for the insertion of spines. These create an undulating upper ridge.

M3l, M4l, M3r and M4r are identical in morphology to the posterior portion of M2. Up to 11 articulation points for spines are present on M3 plates. M4 plates are missing and/or distorted in the holotype but are present in the paratype. One disarticulated plate from the holotype probably corresponds to plate M4l. This also has articulation pits for the spines. M5l and M5r are about half the length of M4, and their posterior part is slightly more curved approaching the articulation with Mp.

Mp is a rectangular plate that is slightly wider than long. It is relatively lower in height than other marginal plates.

A dorsal row of spines lines the marginal frame. These spines articulate along the dorsal edge of the frame from M2 to at least M4 (poor preservation of the posterior frame prevents us from determining if they were also present on M5 and Mp). All the spines have an expanded base and taper distally.

#### Ctenidium and associated plates

The ctenidium is composed of two clearly distinct parts: a dorsal roof of imbricate plates and a ventral row of tooth-like plates. The dorsal part comprises several superimposed series of thin, slightly curved plates that imbricate to the posterior. At the centre, there is a large triangular plate, the suroral (Fig. 4C, F), which roofs the central part of the anterior opening. The dorsal sheet of plates extends laterally to the inflexion point of M2, articulating posteriorly with LL, RL and additional small plates. Because all of these plates overlap without distinct boundaries in the µCT data, they probably formed a single cohesive skirt, with movement of the suroral plate and LL/RL controlling the entire skirt of plates. A row of very small finger-like platelets (only observed directly from the rock: Figure 4H) forms a fringe to the anterior border of the dorsal roofing plates. These do show clear plate boundaries and, therefore, were probably more individually flexible. Immediately to the posterior of the dorsal sheet of ctenidial plates, and lying above the anterior opening, are two pairs of plates (Figure 4A). The inner most of these are small elements that probably articulate with the corners of the suroral plate. These plates connect directly to two much more elongate elements, LL and RL, which form the posterior edge of the dorsal ctenidial sheet. Similar elements are present in *Courtessolea*.

The ventral ctenidium is composed of 14 tooth-like plates. The four central elements are fixed plates that arise as projections from marginal frame plates M0 and M1. The remaining 10 are free plates whose bases fit into sockets along the frontal margin of marginal plates M1 and M2. Ventral ctenidial plates have an expanded base for articulation, a flanged middle and a blade-like distal portion. They imbricate and overlap from posterior to anterior and were clearly highly motile.

#### Integuments

The ventral integument is composed of very thin polygonal plates that form a continuous plated surface. These plates articulate against the ventral edge of marginal plates. The dorsal integument consists of scattered calcified elements and was probably partially uncalcified. The calcified elements are conical with a broad and convex base and a sharp apex. They are circular in outline.

Class **Ctenocystoida** Robison & Sprinkle, 1969 [22].

***Courtessolea moncereti*** Domínguez-Alonso, 1999 [29].

Figure 6

**Types:** Museum National d’Histoire Naturelle, París: holotype, IPM-B 49102.

*Additional material*: MNHN.F.A45783.

#### Locality and horizon

Ferrals de les Montagnes (Montagne Noire, France), *Solenopleuropsis* Assemblage Zone, Lower Languedocian, middle Cambrian, Series 3, Stage 5.

#### Discussion

This taxon was based on a single specimen preserving both part and counterpart. Unfortunately, the posterior part of this specimen is damaged and could not be reconstructed (see [29], p. 211). The new material allows us to identify the periproct position and establish the bilateral symmetry of the thecal plating.

## Discussion

The phylogenetic relationships of extant deuterostomes are now securely founded based on molecular data [4]–[6], which has demonstrated that crinoids are undoubtedly the sister group to other extant echinoderm classes [30]–[32]. However, the phylogenetic position of the extinct asymmetrical fossil echinoderm groups remains disputed (e.g., [12], [20]). This unfortunately hampers our ability to root the echinoderm clade and establish basal morphological and ecological traits, such as which feeding strategy is primitive for the clade. The fossil record of Cambrian echinoderms is very patchy [13], so the few lower Cambrian localities bearing echinoderms do not necessarily capture the earliest history of the group accurately. Nevertheless, some have used stratigraphical arguments to place pentaradiate forms at the base of the echinoderm tree because they appear slightly earlier than other groups in the fossil record [20]. Others have suggested the asymmetric, non-radiate forms are more basal [17]–[19], a view we follow here. Specifically, two groups of highly aberrant, weakly to strongly asymmetric echinoderm, the ctenocystoids and cinctans, have been interpreted as the most basal grades of echinoderms [12], [23].

**Figure 8:**
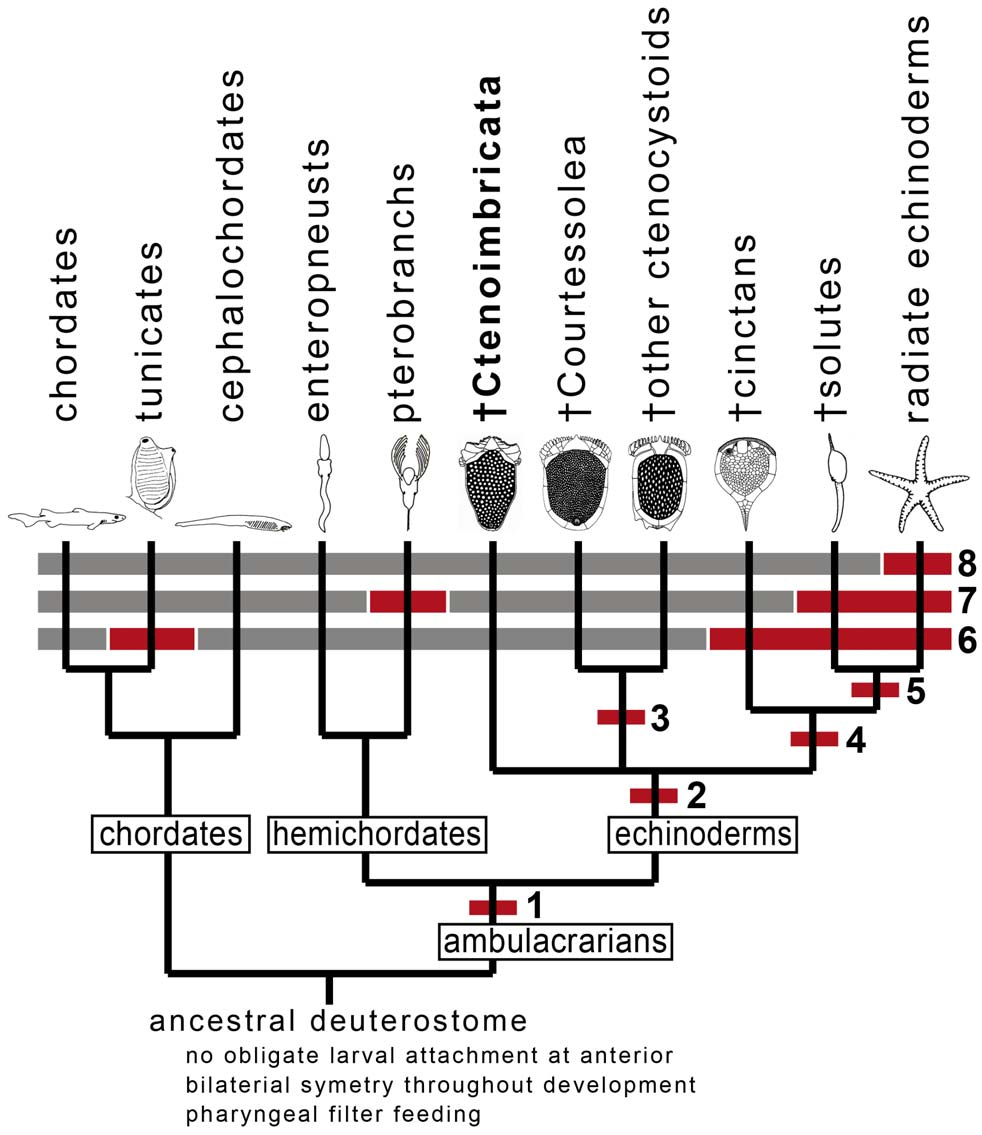
Cladogram showing some major events in deuterostome evolution. Relationships of living organisms are based on molecular data ([4]–[8]); fossils are placed using morphological homologies. 1, Dipleurula-type larva and tripartite organization of body coeloms; 2, Multiplated calcite skeleton with stereom microstructure; 3, Complete ctenidial ring; 4, Periproct non-terminal; 5, Water vascular system with single hydropore (asymmetric development of coeloms); 6, Adult body plan not bilateral (associated with larval attachment and torsion during metamorphosis in extant groups); 7, Tentacular feeding; 8, Radial organization of water vascular system.

The presence of a complete ring of dorsal and ventral ctenoid plates is a synapomorphy *Courtessolea* shares with other, more derived ctenocystoids. *Courtessolea* differs from more derived ctenocystoids, however, in lacking a double marginal ring of plates, and in having a bilaterally symmetrical skeletal frame. It also differs in having a narrow zone of tessellate plates between the dorsal ctenidial plates and the marginal frame plates, which we take to be homologous with the dorsal imbricate plates in *Ctenoimbricata*. We therefore place *Courtessolea* as sister group to all other ctenocystoids but more derived than *Ctenoimbricata. Ctenoimbricata* shares clear homologies with both ctenocystoids and cinctans as discussed above. All three have a frame of marginal ossicles encircling dorsal and ventral plated membranes. In *Ctenoimbricata*, Cincta and *Courtessolea* this frame is single, but in more derived ctenocystoids it is double and asymmetric. In Cincta the frame is also asymmetrical and extends to the posterior as a long stabilizing bar, an autapomorphy of that group. The periproct is posterior in *Ctenoimbricata*, *Courtessolea* and Ctenocystoidea, but opens through the dorsal membrane in the former two and through the marginal frame in ctenocystoids. In Cincta the periproct pierces the dorsal membrane, but is displaced to the left anterior indicating a U-shaped digestive tract. *Ctenoimbricata* has only spinose ctenidial elements ventrally and has a labral-like sheet of plates dorsally. *Ctenoimbricata* is thus more basal than *Courtessolea*, being either sister group to the Ctenocystoidea or sister group to all echinoderms. Figure 8 summarizes the phylogenetic position of these primitive echinoderms with respect to other deuterostomes.

We show here that both *Ctenoimbricata* and *Courtessolea* have a bilateral body plan with an anterior mouth and posterior anus defining an anterior–posterior body axis, a bilateral arrangement of ctenoid plates, and symmetrically arranged plating along each side of the frame. These therefore provide an important insight into the form and functional biology of the earliest stem group echinoderms, prior to the acquisition of radiality or even asymmetry. Interestingly, evidence points to a bilateral body plan being widespread amongst primitive ctenocystoids. For example, restudy of the type material of *Jugoszovia* from Poland reveals for the first time that it is also bilateral, as may be an undescribed ctenocystoid from Prague figured by Fatka and Kordule ([33], plate 1 fig. 3) and wrongly placed within *Etoctenocystis*. The original specimen and more than 50 new specimens (Zamora and Fatka unpublished) reveal that its ventral frame is bilateral with a Mp tapering the posterior side of the marginal ring.

As well as allowing well-founded homologies to be drawn between cinctans and ctenocystoids, these fossils reveal two important features about the evolutionary history of early deuterostomes (Figure 8). First, both hemichordates and echinoderms diversified very early in their history to give rise to sister clades with convergent feeding strategies, either using tentacles for suspension feeding (pterobranchs, ambulacral-bearing echinoderms) or pharyngeal filtering for deposit feeding (enteropneusts, cinctans/ctenocystoids), the latter being primitive as previously hypothesized [34]. Second, it is noteworthy that torsion and a striking deviation from bilaterality have occurred independently in tunicates and within echinoderms, and in both cases are associated with the adoption of attachment [10].

## Supporting Information

Movie S1 ***Ctenoimbricata spinosa*** **gen. et sp. nov.** Movie showing the olotype (MPZ 2011/93) and a computer reconstruction of this specimen in plan view.
(AVI)

Movie S2 ***Ctenoimbricata spinosa*** **gen. et sp. nov.** Movie showing a computer reconstruction of the holotype (MPZ 2011/93) in lateral view.
(AVI)
